# ESBL colonization and acquisition in a hospital population: The molecular epidemiology and transmission of resistance genes

**DOI:** 10.1371/journal.pone.0208505

**Published:** 2019-01-14

**Authors:** Stefan Hagel, Oliwia Makarewicz, Anita Hartung, Daniel Weiß, Claudia Stein, Christian Brandt, Ulrike Schumacher, Ralf Ehricht, Vladimir Patchev, Mathias W. Pletz

**Affiliations:** 1 Institute of Infectious Diseases and Infection Control, Jena University Hospital, Jena, Germany; 2 Infectognostics Research Campus, Jena, Germany; 3 Abbott (Alere Technologies GmbH), Jena, Germany; 4 Center for Clinical Studies, Jena University Hospital, Jena, Germany; University Hospital Basel, SWITZERLAND

## Abstract

A prospective cohort study (German Clinical Trial Registry, No. 00005273) was performed to determine pre-admission colonization rates, hospital acquisition risk factors, subsequent infection rates and colonization persistence including the respective molecular epidemiology and transmission rates of extended-spectrum β-lactamase (ESBL)-producing *Enterobacteriaceae* (EPE). A total of 342 EPEs were isolated from rectal swabs of 1,334 patients on admission, at discharge and 6 months after hospitalization. Inclusion criteria were patients’ age > 18 years, expected length of stays > 48 hours, external referral. The EPEs were characterized by routine microbiological methods, a DNA microarray and ERIC-PCR. EPE colonization was found in 12.7 % of admitted patients, with the highest rate (23.8 %) in patients from nursing homes. During hospitalization, 8.1 % of the patients were *de novo* EPE colonized, and invasive procedures, antibiotic and antacid therapies were independent risk factors. Only 1/169 patients colonized on admission developed a hospital-acquired EPE infection. *Escherichia coli* was the predominant EPE (88.9 %), and 92.1% of the ESBL phenotypes could be related to CTX-M variants with CTX-M-1/15 group being most frequent (88.9%). A corresponding β-lactamase could not be identified in five isolates. Hospital-acquired EPE infections in patients colonized before or during hospitalization were rare. The diversity of the EPE strains was much higher than that of the underlying plasmids. In seven patients, transmission of the respective plasmid across different species could be observed indicating that the current strain-based surveillance approaches may underestimate the risk of inter-species transmission of resistance genes.

## Introduction

Healthcare- and community-associated infections with extended-spectrum β-lactamase (ESBL)–producing *Enterobacteriaceae* (EPE) have become common globally and are associated with increased mortality as well as hospital stays of excessive length and cost compared with infections with susceptible strains.[1]

Since carbapenems are considered the best treatment for EPE infections, the spread of EPEs has resulted in the increased use of carbapenems with the consequent emergence and dissemination of carbapenemase-producing enterobacteria.[2] Despite increasing concerns regarding EPEs, their transmission inside and outside healthcare institutions is still incompletely understood, resulting in controversy regarding the appropriate recommendations for infection control measures in clinical settings.[3] During the past decade, several surveys have addressed EPE transmission and colonization as well as their associated infections in cohorts with different medical conditions to discern patient-related and iatrogenic factors that might facilitate these processes.[4–6] However, very few longitudinal studies examining the duration of colonization were supported by molecular typing to differentiate from *de novo* colonization by another EPE strain.[7–9]

The aims of the present study were as follows: (i) to determine the baseline prevalence of EPEs at hospital admission; (ii) to monitor intra-hospital EPE infection and intra-hospital transmission rates, including associated risk factors; (iii) to monitor patient colonization status at hospital discharge and (iv) at a 6 month-follow-up in colonized patients; and (v) to define the molecular characteristics of all isolated EPEs at the strain, plasmid and resistance gene levels.

## Materials and methods

### Study setting and participants

This prospective observational study was conducted at the Jena University Hospital between 23^rd^ September 2013 and 30^th^ September 2015. It was approved by the ethic committee of the Jena University Hospital (3852/07-13) and registered in the German Clinical Trial Registry (00005273). Patients were screened for EPE colonization within 48 hours of admission (T0) to the Departments of Cardiothoracic Surgery (CTS), Gastroenterology-Hepatology-Infectology (GHI) and Geriatric Medicine (GM). Inclusion criteria were age ≥ 18 years, expected length of stay > 48 hours and external referral. In accordance with national guidelines, patients colonized with EPE did not undergo single-room isolation, except for those with fluoroquinolone-resistant EPE in the intensive and intermediate care units (ICU and IMC). A second screening was performed at hospital discharge (T1). Patients who tested positive at discharge underwent a third sampling at 6 months after discharge (T2), provided that informed written consent was given.

### Collection and processing of samples

EPE were selectively isolated from rectal swabs (in Amies medium) on CHROMagar ESBL/KPC plates (Mast Diagnostica). Species and resistance phenotypes were routinely identified using VITEK2 (bioMérieux). DNA was isolated using the Qiagen DNeasy Blood & Tissue Kit (Qiagen).

### Molecular genotyping

Microarray genotyping was performed using the CarbDetect AS-2 Kit and the ArrayMate Reader (Alere Technologies) as described previously.[10] Briefly, 62 different β-lactamase variants, 56 other resistance and virulence determinants, and 13 genus- and species-specific genes were assessed.

To assess clonality of the *E*. *coli* isolates (n = 301), ERIC-PCR was performed using the primers ERIC1 and ERIC2 as described previously.[11] ERIC-PCR was not performed for other species because the differentiation power of the ERIC-primers in these species is not well validated.

### Variables and statistics

The EPE colonization rate at the time of admission, the rate of EPE infections during hospital stay, the proportion of patients at discharge colonized by an EPE, the persistence rate after 6 months and the clonality and resistance pattern of the isolates were assessed. Co-variables such as reasons for admission, demographics, antibiotic and antacid therapy or invasive procedures were documented (Tables A-C in S1 File). The data were collected in OpenClinica Community edition, version 3.0.4 (OpenClinica, LLC). Descriptive statistical analysis was performed with SAS 9.4 (SAS Institute, Inc).

The ERIC-PCR patterns were compared by applying the Dice similarity coefficient (0.5 % optimization, 1 % tolerance), and the binary microarray pattern was analysed via the Pearson correlation and chi-square test, all of which were performed using BioNumerics 7.6 (Applied Math NV). Phylogenetic tree construction and clustering were performed using the unweighted pair group method with arithmetic means (UPGMA). Ward’s method was used to reduce the number of microarray clusters for the chi-square test. Due to the low number of detected isolates, descriptive statistics could not be performed for the follow-up group.

## Results

### ESBL colonization rates

**Fig 1:**
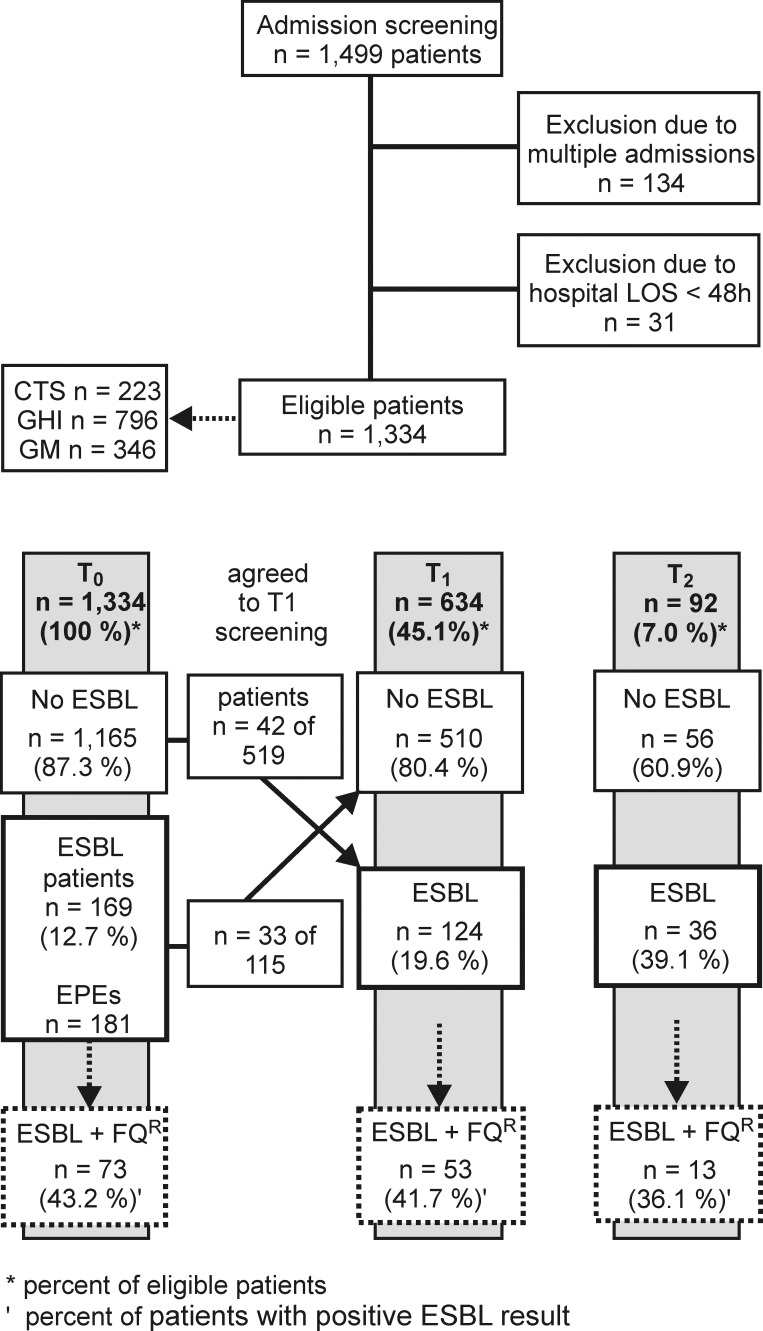
Study design and recruitment. T0, admission; T1, discharge from the hospital; T2, follow-up after 6 months; *, percent of eligible patients; FQ^R^ fluoroquinolone resistant; and ‘, percent of EPE-colonized patients.

On admission (T0), 169/1,334 patients (12.7 %) were colonized by EPE (Fig 1). The highest EPE rate (23.8 %) was found in patients from nursing homes. Rectal screening at discharge from the hospital (T1) could not be performed for 700 patients, including 54 patients who tested positive on admission, due to withdrawn consent. In 33/115 patients (28.7 %) who tested positive on admission, no EPE could be detected at discharge. In total, EPEs were isolated from 124/634 (19.6 %) patients at T1 with 42/519 patients (8.1 %) newly colonized by an EPE (negative at T0) during hospitalization (Fig 1). The median time interval between admission and discharge was 8 days (mean 11.5 ± 11.9 days). Of the 124 patients who tested positive at T1, 92 gave consent for a follow-up examination (T2) (mean time to follow-up 213.8 ± 41.0 days); of those, 36 patients (39.1 %) were positive for EPEs. Characteristics of the three groups (T0, T1 and T2) are given in the Supplement (Table A-C in S1 File).

### Risk factor analysis and infection rates

Analysis of socio-demographic factors and medical history (Tables D-F in S1 File) revealed that electively admitted patients had a lower risk of being colonized with EPE on admission compared to patients admitted emergently, whereas a prior ICU stay (within the last 6 months) showed a trend towards enhanced risk of EPE presence. Colonization on admission was not influenced by other variables.

The relative risk of EPE colonization during the hospital stay was notably higher for patients who were admitted to the surgical department, who underwent invasive procedures (except for endoscopy), and who received antibiotics and/or proton-pump inhibitors (PPI). A significant association of *de novo* colonization with use of a defined class of antibiotics was not observed. The factors ‘male sex’ and ‘surgical indication during the preceding hospitalization’ displayed pronounced but non-significant trends towards increased risk of persistent ESBL colonization after discharge. No effect was observed for the factors ‘recurrent hospitalization’ or ‘antibiotic treatment’.

During hospitalization, only one of the 169 patients colonized at admission acquired an infection caused by an EPE (0.6 %). In this geriatric female patient, the presence of ESBL-producing and ciprofloxacin-resistant *Escherichia coli* and *Klebsiella pneumoniae* was documented in urine and faecal samples on admission, with subsequent isolation of *K*. *pneumoniae* in a blood culture sampled during an episode of central line-associated bloodstream infection.

### Colonizing EPE species

In total, 342 EPE isolates were collected from all patients (S1 Table). No isolate with carbapenem resistance was found. In 13 patients, more than one EPE isolate or species were collected at one timepoint. *E*. *coli* was the most frequently detected EPE overall, accounting for more than 88.9 % of the isolates, followed by *K*. *pneumoniae* (8.2 %); other *Enterobacteriaceae* were present in individual cases (Table 1). There were no significant differences (p-value > 0.5) in the distributions of the species between the different sampling time points.

**Table 1:** Prevalence of EPE species at admission (T0), discharge (T1) and follow-up (T2) and of newly acquired EPE colonization at T1.

	All at admission (T0)	All at discharge (T1)	Newly acquired at T1^A^	All at follow-up (T2)	Total
***E*. *coli***	156 (86.7%)	113 (89.7 %)	40 (88.9 %)	35 (97.2 %)	304 (88.9 %)
***K*. *pneumoniae***	20 (11.1 %)	7 (5.6 %)	0	1 (2.8%)	28 (8.2 %)
***K*. *oxytoca***	2 (1.1 %)	1 (0.8 %)	1 (2.2 %)	0	3 (0.87)
***C*. *freundii***	0	3 (2.4 %)	2 (4.4 %)	0	3 (0.9 %)
***E*. *cloacae***	2 (1.1 %)	2 (1.6 %)	2 (4.4 %)	0	4 (1.17 %)
**Total**	180 (100 %)	126 (100 %)	45 (100 %)	36 (100 %)	342 (100 %)

^A^Patients with newly acquired EPE colonization represent a subgroup of T1.

### Frequency of β-lactamase genes

In 315/342 (92.11 %) of the isolates, the ESBL phenotype could be assigned to CTX-M variants. Further analysis of the resistance patterns revealed that the vast majority of the isolates bore β-lactamases of the CTX-M-1/15 group, followed by TEM. The frequency of other ESBLs was below 10.0 % (Table 2). The CTX-M1/15 group was stronger related to *E*. *coli*, while SHV was predominantly found in *K*. *pneumoniae*, but the CTX-M-9 and TEM groups were distributed equally between both species (Table G in F1 File). None of the isolates bore two different CTX-M groups, but the co-existence of a CTX-M-1/15 group with a TEM group was frequent and predominantly found in *E*. *coli* isolates (Table H in S1 File); however, all 12 EPEs harbouring CTX-M1/15-like and SHV β-lactamases were *K*. *pneumoniae*. The rates of the CTX-M-1/15 and CTX-M-9 groups varied slightly and inversely, with reduced rates of CTX-M-1/15 and increased rates of CTX-M-9 groups at 6 months after discharge compared to those at T0 and T1. The CTX-M-1/15-like rates were highest and the CTX-M-9-like rates lowest in the sup-population of patients who acquired a new EPE in the hospital (Table 2). The ESBLs of the CTX-M-2 and CTX-M-8 groups were found only in individual isolates. Additionally, a VEB-like class A β-lactamase was present in an *E*. *coli* isolate together with a CTX-M-1/15-like ESBL at T0. Other class A β-lactamases covered by the array (PER-like and SME-like) were not identified.

**Table 2:** Prevalence of the different β-lactamase genes in EPE isolates at admission (T0), discharge (T1) and follow-up (T2), as well as in the newly acquired EPE colonization isolates at T1.

	All at admission (T0)	All at discharge (T1)	Newly acquired at T1^A^	All at follow-up (T2)	Total
**CTX-M-1/15**	146 (81.1%)	106 (84.1 %)	40 (88.9%)	27 (75.0 %)	279 (81.2 %)
**TEM**	93 (51.7 %)	59 (46.8 %)	16 (35.6 %)	18 (50.0 %)	170 (49.7 %)
**CTX-M-9**	18 (10.00 %)	9 (7.1 %)	2 (4.4 %)	5 (13.9 %)	32 (9.4 %)
**OXA-1**	15 (8.3 %)	11 (8.7 %)	2 (5.1 %)	2 (5.6 %)	28 (8.2 %)
**SHV**	15 (8.3 %)	9 (7.1 %)	1 (2.2 %)	2 (5.6 %)	26 (7.6 %)
**OXA-2**	10 (5.6 %)	7 5.6 %)	2 (4.4 %)	2 (5.6 %)	19 (5.6 %)
**OXA-10-like**	5 (2.8 %)	1 (0.8 %)	0	0	6 (1.8 %)
**OXA-60-like**	2 (1.1 %)	1 (0.8 %)	0	0	3 (0.8 %)
**CTX-M-2**	1 (0.6 %)	1 (0.8 %)	0	0	2 (0.6 %)
**CTX-M8**	1 (0.6 %)	1 (0.8 %)	0	0	2 (0.6 %)

^A^The newly acquired EPE colonization β-lactamase genes represent a subgroup of T1.

The most frequent OXA groups were OXA-1-like, identified in 10/20 *K*. *pneumoniae* but only in 18/304 *E*. *coli* isolates, and OXA-2-like, found in 18/304 *E*. *coli* and 1/3 *C*. *freundii* isolates. The OXA-10 and OXA-60 groups were solely identified only in *E*. *coli* isolates. Other OXA ESBL-groups were not identified.

The class C β-lactamase CMY was identified in 2/3 *C*. *freundii* isolates only at T1, and MIR was found in 4/304 *E*. *coli* isolates from different timepoints. A FOX class C β-lactamase was identified in one *E*. *coli* isolate at T0. Other class C β-lactamases were not found.

In five isolates (1.5 %), including one *E*. *coli*, one *K*. *pneumoniae* and three *E*. *cloacae*, a corresponding β-lactamase could not be identified by the microarray-based assay. Genes encoding carbapenemases (21 genes in total, including OXA-48, KPC and metallo-β-lactamases) were not detected.

### Frequency of other resistance associated genes

Concomitant phenotypic resistance to ciprofloxacin was documented in less than half of the isolates (Fig 1). Molecular analysis revealed that in 11.6 % of the isolates at both T0 and T1 and 13.8 % at T2, fluoroquinolone resistance might be related to the *qnrA1* (in *E*. *coli*), *qnrB* (in *K*. *pneumoniae*) and *qnrS* (in both species) genes, all of which encode plasmid-associated gyrase-protective proteins.

**Fig 2:**
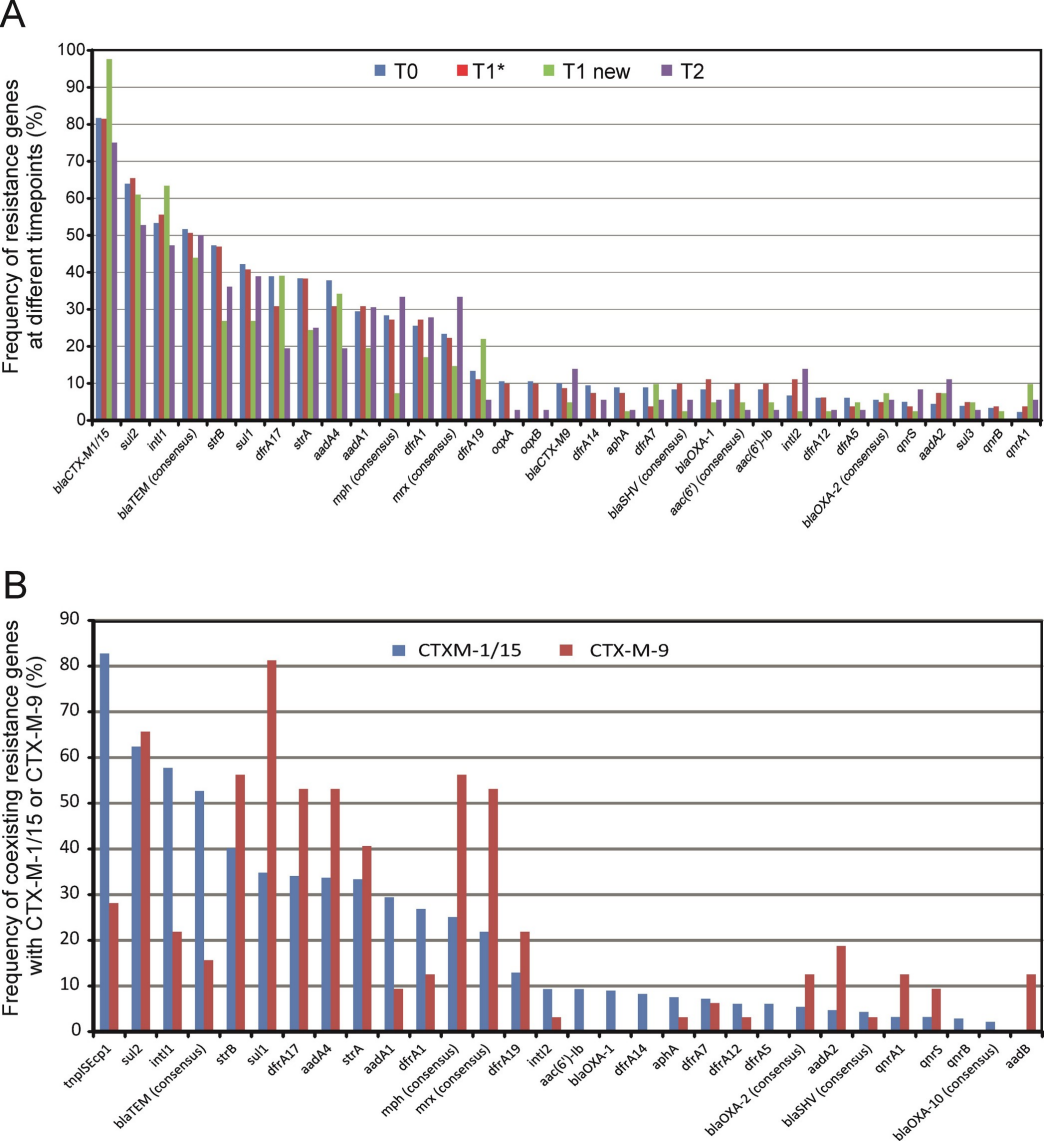
Distribution of different resistance genes and related determinants in the isolates obtained in this study. (A) Frequency of the resistance genes with an overall occurrence of at least 5 %, sub-grouped as follows: T0 = all EPE isolates at admission (n = 180, 100 %), T1* = EPE isolates at discharge with a positive T0 screening (n = 81, 100%), T1 new = isolates from patients who acquired an EPE during their hospital stay (n = 45, 100 %), and T2 = all isolates at follow-up (n = 36, 100 %). (B) Frequency of coexisting resistance patterns with CTX-M1/15 or CTX-M-9 group ESBLs. The rates of the alleles were calculated relative to the total number (100 %) of isolates in each respective subgroup. Molecular analysis was performed using the CarbDetect AS-2 Array. *strA* and *strB* genes encode phosphotransferases, *sul* genes encode dihydropteroate synthase, *dfrA* alleles encode dihydrofolate reductase, *aadA* alleles encode aminoglycoside adenyl-transferase, *aac*(6’) alleles encode aminoglycoside acetyltransferase, *mph* encodes macrolide 2'-phosphotransferase, *mrx* encodes an unknown protein, the *qnr* genes encode gyrase-protective proteins, and the operon *oqxAB* encodes an efflux pump.

The most frequent genes associated with aminoglycoside resistance in all isolates were *strA* (34.0 %) and *strB* (43.2 %) (Fig 2A). These genes usually occur as a pair; however, in 18.4 % of all *strB*-positive isolates, *strA* was missing. The most frequently found genes associated with sulfonamide and trimethoprim resistance were *sul2* (61.6 %) and *sul1* (38.7 %), as well as different *dfr* alleles, predominantly *dfrA17* (34.0 %), *dfrA1* (24.6 %) and *dfrA19* (12.9 %), all of which are associated with integron structures and plasmids [12, 13]. The *sul3* gene was occasionally identified. Other detected genes frequently associated with resistance to aminoglycosides that are often present on mobile elements were *aadA4* (32.6 %) and *aadA1* (28.2 %). Signatures of mobile elements frequently found in this study were *tnpISEcp1* (71.6 %) that was first described in connection with the CTX-M dissemination [14], and *intI1* (53.4 %), which is responsible for the dissemination of resistance genes in Gram-negative bacteria; both are also commonly plasmid-located and linked to each [12].

In general, the pattern of the resistance genes that were associated with the CTX-M-1/15-like or the CTX-M-9-like variants differed significantly (p-value > 0.0001) (Fig 2B), suggesting that different plasmid structures are associated with these prevalent CTX-M groups. Among the 279 isolates bearing an ESBL of the CTX-M-1/15 group, 231 (82.8 %) and 161 (57.7 %) co-existed with *tnpISEcp1* and *intI1*, respectively, but only 9 (28.13 %) and 7 (21.9%) of the 32 CTX-M-9 group-carrying isolates did. The *intI2* was identified in only 27/342 (7.9 %) isolates, all but one of which carried CTX-M-15/1 group ESBLs; *intI3* was not detected in any of the isolates. The CTX-M-9 group was strongly related to the *sul1* gene (81.3%), followed by the *strB* (56.3 %), *mph* (56.3 %) and *mrx* (53.1 %) genes associated with macrolide resistance, as well as *dfrA17* and *aadA4* (53.1% each).

### Plasmid traces

Co-colonization by different EPE species was observed in 6 patients: 5 by *E*. *coli* and *K*. *pneumoniae* and one by *E*. *coli* and *K*. *oxytoca*. The resistance gene patterns between *E*. *coli* and either *K*. *pneumoniae* or *K*. *oxytoca* were similar in two cases. Additionally, in one patient (of four cases) in whom the EPE species found at T0 was replaced by another EPE species at a later timepoint, the resistance gene pattern was similar between *E*. *coli* (T0) and *C*. *freundii* (T1). All seven cases indicated interspecies plasmid transfer.

### Differences between admission and hospital-acquired EPE

**Fig 3:**
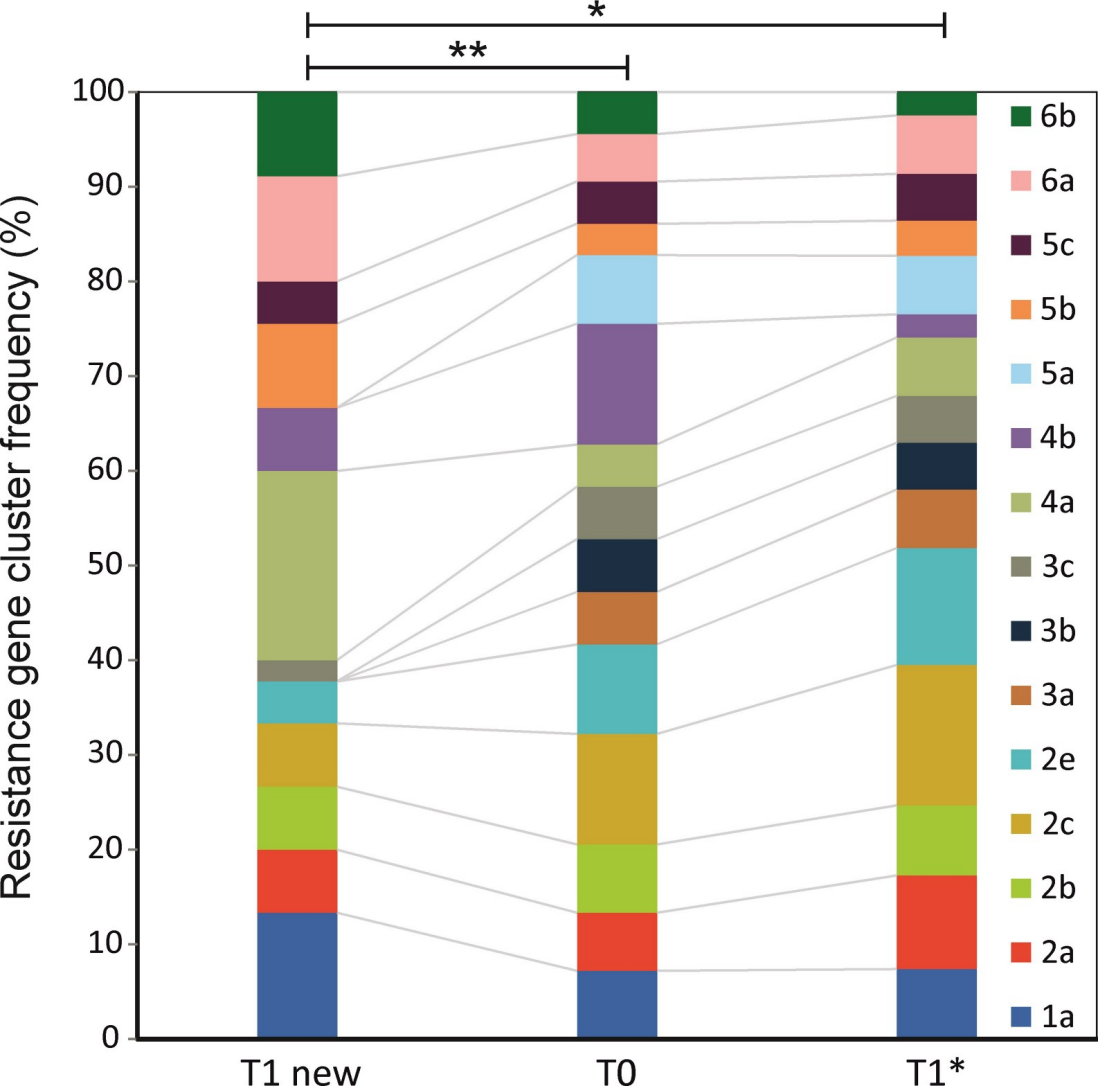
Distribution of the ward clusters calculated for the plasmid-associated resistance patterns in patients at admission (T0) and discharge for those who were colonized during hospitalization (T1 New) and for those already positive at admission (T1*). The significance of the differences was assessed by Pearson's chi-square test.

The clonality of the plasmid-associated resistance patterns was assessed by UPGMA and Ward cluster analysis and the lineages were subgrouped resulting in 66 UPGMA but 15 Ward-subgroups. To investigate whether there were preferences in the plasmid-associated pattern in *de novo* colonized patients, we performed a contingency analysis for the Ward-clustered microarray pattern (Fig A in S1 File). No significant differences between the EPEs at T0 and T1 (P-value 0.7899) were revealed, but the microarray patterns differed significantly between patients who acquired an EPE *de novo* in the hospital and T0 (p-value 0.005) (Fig 3) with five clusters, all of which carried the *tnpISEcp1* element that was more dominant in the hospital-acquired group. The largest of these clusters (20 %) was distinguished by the presence of the CTX-M-1/15 group, *dfrA17*, *aadA4* and *sul2*, suggesting type 1 integron cassette with an array of *int1*-*dfrA17*-*bla*_*CTX*-M-1/15_-*aadA4*-*sul2*.

### Clonality and transmissions

In 96.1% of the patients with positive screening at T0 and T1, or T1 and T2, the isolated species were identical. The clonality of the isolates obtained from each of those patients was assessed by a cluster analysis of the ERIC-PCR, which has been shown to exhibit sufficient differentiation power for molecular typing of *E*. *coli*.[11] The ERIC-PCR band pattern strongly varied throughout the 301 *E*. *coli* isolates. The lineages were subgrouped based on similarity > 90 % (S1 Fig) resulting in 50 subgroups.

The ERIC-PCR patterns indicated lower similarity compared to the UPGMA subgroups of the plasmid-associated resistance patterns at the different timepoints in the patients. The highest similarity was found between the admission and discharge samples (ERIC-PCR 38.6 %, microarray 67.9 %), and the lowest was observed between the admission and follow-up samples (21.4 % vs 40.7 %, respectively) (Table 3).

**Table 3:** Similarity of the plasmids and isolates between the different screening time points or within a single time point (TX).

	All isolates		*E*. *coli* isolates only		
Timepoints	Number (n) of similar plasmid patterns (%)	Number (n) of all Cases	Number (n) of similar RAPD patterns (%)	Number (n) of co-similar plasmid and RAPD patterns^C^ (%)	Number (n) of all cases
**TX**^A^	10 (76.9 %)	13	3 (33.3%)	2 (22.2%)	9
**T0/T1**^B^	53 (67.9%)	78	28 (38.6 %)	15 (20.5 %)	73
**T1/T2**^B^	10 (34.5 %)	29	7 (25.9 %)	6 (22.2%)	27
**T0/T2**^B^	11 (40.7 %)	27	6 (21.4 %)	4 (14.3 %)	28
**T0/T1/T2**	8 (34.8 %)	23	2 (9.1 %)	2 (9.1 %)	22

^A^TX, cases with multiple isolates of the same species at a given time point;

^B^cases where all three screenings were collected (T0/T1/T2);

^C^co-similar plasmid and RAPD patterns in one isolate.

Assuming that transmission may occur only between patients who were hospitalized in local (same ward), temporal and chronological proximity, we compared the clonal subgroups to estimate probable transmissions between the patients: UPGMA subgroups for strain clonality and Ward subgroups for resistance because these were more robust in case of multiple plasmids per cell. In four cases, transmission could not be excluded, as the donor and recipient were hospitalized during overlapping periods in the same ward. All donors were EPE colonized on admission. In three of these cases, the EPE was identified in a newly colonized patient at discharge. These patients were found to be carrying a plasmid belonging to Ward-cluster 4a that was predominantly associated with *de novo* acquisition in the hospital. In one case, the EPE previously found at admission was replaced in the recipient by another EPE brought into the hospital by the donor.

## Discussion

The prevalence of EPE colonization at admission in our study was twice as high as the reported 6.03 % in 2014 for another German cohort and was attributed to the high average age of our cohort, which has been repeatedly emphasized as a risk factor for EPE colonization. [8, 15–17] In line with earlier findings, the rate of EPE carriage was highest in patients residing in nursing/retirement homes due to the generally increased EPE prevalence reported in these environments.[18, 19] In accordance with some studies, the rate of in-hospital EPE acquisition was 8.09 %,[20] but in nearly 1/3 of the patients colonized on admission, EPE was not found after hospitalization. It is unclear whether the EPE was lost or whether the detection method was insufficient.

With only one case, the risk for bloodstream infections due to EPE colonization was substantially lower in our cohort (0.6 %) compared to reports on patients admitted to haematology/oncology departments.[20, 21] To our knowledge, this study investigated the epidemiology of EPE colonization and subsequent infection in a cohort without increased risk (non-haematological or neonatal patients) for the first time; thus, we cannot directly compare the results. However, our results suggest that covering EPEs with an empiric antibiotic treatment in case of hospital-acquired infections of EPE-colonized patients may be required only for severely immunocompromised patients.

In agreement with other studies reporting a median clearance time of 6.6 months for EPE after hospitalization,[7] we detected EPE colonization in nearly half of the follow-up patients.

The most frequently reported risk factors for EPE colonization and infection at hospital admission are almost the same as those during hospitalization, i.e., advanced age, long hospital and/or ICU stay, invasive medical procedures, and antibiotic treatment.[20] In line with other recent studies, we also identified that PPIs increase the risk of hospital-acquired EPE colonization.[6, 22] PPI treatment has been hypothesized to facilitate the transition of EPE to the gut microbiome via oral intake.

In accordance with other studies, *E*. *coli* was the predominant EPE and the CTX-M-1/15 group was the predominant ESBL independent of the timepoint.[6, 22] In our study, CTX-M-1/15-like ESBLs were strongly related to IS*Ecp1* and class 1 integrons, the *sul1* and *sul2* genes and *str* alleles, and the TEM β-lactamase group. CTX-M ESBLs have been frequently found in IS*Ecp1* transposition units in *Enterobacteriaceae*,[23–25] as well as in association with class 1 integrons that often carry *sul1* genes,[26, 27] while *sul2* genes are associated with large plasmids.[28] Other clinically relevant resistance genes with high prevalence included the plasmid-associated *dfr* and *aadA* alleles that are all known to form gene cassettes in class 1 integrons spreading among *Enterobacteriaceae* as well as *Pseudomonas aeruginosa* and *Acinetobacter* spp.[29] The co-existence of several different integron-related genes suggests a co-localization of several plasmids that can mobilize resistance genes, enhancing their intra- and interspecies mobility and transferability, which may explain the identical plasmid-associated molecular patterns observed in different species in seven patients.

Nearly half of the EPEs were concomitantly resistant to fluoroquinolones. We did not analyse the mutations within the *gyr* and *par* genes that are known to confer high-level fluoroquinolone resistance, but in one tenth of the EPEs, the identified *qnr* genes, which can be transferred by IS*Ecp1* elements,[24] might contribute to this resistance.

The differences found in plasmid-associated patterns between hospital-colonized patients and those colonized at admission suggested that some ESBL-carrying plasmids might be more related to hospital environments than others. This particularly applies to those with the CTX-M-1/15-like ESBLs. Conversely, the higher rate of CTX-M-9-like ESBLs observed in the follow-up screening suggests that EPEs bearing such plasmids persist longer. Even if transmission from colonized patients to other patients was rare, it might be under-estimated in our study due to the limited number of study subjects. Three of the four supposed transmissions seemed to be associated with a specific plasmid; thus, a hospital source cannot be excluded.

In summary, the present study found a very low rate of EPE bloodstream infections compared to studies in patients in haematology/oncology departments and confirmed some known risk factors associated with pre-hospital EPE colonization and intra-hospital EPE acquisition. CTX-M-1/15-like ESBLs were the predominant variants associated with highly variable multidrug resistance plasmids that were obviously exchanged between different species. In general, plasmid transfer between Gram-negative species must be expected and therefore, the current strain-based surveillance approach may underestimate the transmission of resistance genes tween the species.

## Supporting information

S1 File Supporting tables and figures to the manuscript. (PDF)

S1 Table Details of the isolates. (CSV)

S1 Fig Phylogeny of the *E*. *coli* isolates based on the ERIC-PCR band pattern. Analysis was performed applying the Dice similarity coefficient (0.5% optimization, 1% tolerance) using BioNumerics 7.6 software (Applied Math NV, Sint-Martens-Latem, Belgium). Tree construction was performed using the unweighted pair group method with arithmetic means (UPGMA).
(JPG)
