# Supplementary material for: ESBL colonization and acquisition in a hospital population: The molecular epidemiology and transmission of resistance genes
Source: PLoS One. 2019 Jan 14;14(1):e0208505. doi: 10.1371/journal.pone.0208505 (PMC6331103; doi:10.1371/journal.pone.0208505)
Supplement: S1 File — (PDF) [file pone.0208505.s001.pdf]

S1 File. Supporting Tables and Figures to the manuscript.

ESBL colonization and acquisition in a hospital population: the molecular epidemiology and transmission of resistance genes.

Stefan Hagel[1*], Oliwia Makarewicz[1,4*], Anita Hartung[1], Daniel Weiß[1,3,4], Claudia Stein[1,4]; Christian Brandt[1,4], Ulrike Schumacher[2], Ralf Ehricht[3,4], Vladimir Patchev[1,2], Mathias W. Pletz[1,4#]

[1] Institute of Infectious Diseases and Infection Control, Jena University Hospital, Jena, Germany

[2] Center for Clinical Studies, Jena University Hospital, Jena, Germany

[3] Abbott (Alere Technologies GmbH), Jena, Germany

[4] Infectognostics Research Campus, Jena, Germany

Table A. Characteristics of patients at admission (T0) (n = 1,334)

Variable	n	% from total
Age (years; mean ± S.D.)	67.6 ± 15.9	
Sex		
• male	664	49.8
• female	670	50.2
Department		
• Cardiothoracic Surgery	219	16.4
• Gastroenterology, Hepatology and Infectious Diseases	770	57.7
• Geriatric Medicine	345	25.9
Admission type		
• elective	1181	88.5
• emergency	152	11.4
• unknown	1	0.1
Indication		
• surgical	214	16.0
• non-surgical	1115	83.6
• unknown	5	0.4
Place of residence		
• own dwelling	991	74.3
• nursing home	42	3.1
• rehabilitation facility	288	21.6
• unknown	13	1.0
Hospitalization during last 6 months		
• no	311	23.3
• yes	804	60.3
• unknown	219	16.4
ICU stay during last 6 months		
• no	645	48.4
• yes	65	4.9
• unknown	624	46.8
Antibiotic use during last 3 months		
• no	157	11.8
• yes	237	17.8
• unknown	940	70.5

Table B. Comparative characteristics of patients colonized *de novo* with ESBL during hospital stay (n = 42) and subjects who tested pathogen-free at admission and discharge (n = 477).

Variable	ESBL *de novo*-colonized		ESBL-free	
	n	% from subgroup	n	% from subgroup
Age (years; mean ± S.D.)	68.4 ± 14.5		66.8 ± 16.2	
Sex				
• male	16	38.1	213	44.7
• female	26	61.9	264	55.3
Admission type				
• elective	40	95.2	430	90.1
• emergency	2	4.8	47	9.9
Primary indication				
• surgical	6	14.3	25	5.2
• non-surgical	36	85.7	448	93.9
• unknown			4	0.8
Invasive procedures (cases)*				
• diagnostic endoscopy	24	52.2	303	63.5
• surgery (including endoscopic surgery)	21	45.6	184	38.6
• other (invasive respiration, i.v. line, catheter, parenteral nutrition)	19	41.3	62	13.0
ICU admission (cases)	7	16.7	31	6.5
Average ICU stay (days; mean ± S.D.)	6.8 ± 4.2		4.9 ± 2.6	
Antibiotic use (cases)*				
• none	25	59.5	379	79.5
• Cephalosporins 1st generation	4	23.5**	21	21.4**
• Cephalosporins 2nd generation	2	11.8**	5	5.1**
• Cephalosporins 3rd generation	3	17.6**	14	14.3**
• Fluoroquinolones	6	35.3**	25	25.5**
• Penicillins with BL inhibitor	4	23.5**	22	22.4**
• Penicillins without BL inhibitor	-		2	2.0**
• Carbapenems	-		7	7.1**
• Other (aminoglycosides, glycopep-tides, macrolides, cotrimoxazole)	14	82.3**	52	53.1**
Antacid therapy (cases)	31	73.8	275	57.8

**) individual subjects may have received more than one procedure/treatment/drug class*

***) percentage rates represent proportions of subjects having received any antibiotic treatment*

Table C. Characteristics of patients diagnosed with persistent ESBL pathogens (n = 36) after follow-up for 6 months upon hospital discharge.

Variable	n		% from subgroup	
Age (years; mean ± S.D.)	67.7 ± 13.5			
Sex<br>• male<br>• female	 23<br>13		 63.9<br>36.1	
	yes	unknown	yes	unknown
Hospitalization during follow-up period (cases)	13	10	36.1	27.8
Antibiotic treatment during follow-up (cases)	9	26	25.0	72.2
ICU admission during follow-up (cases)	1	11	2.8	30.6

Table D. Influence of demographic factors and medical history on the prevalence of EPE-colonization in patients at admission (T0).

Factor	RR	95 % confidence intervals
Male sex	1.177	0.887 – 1.562
Type of admission (elective vs. emergency)	0.648	0.448 – 0.937
Type of indication (surgical vs. non-surgical)	0.821	0.542 – 1.243
Preceding stay in hospital	0.946	0.692 – 1.294
Preceding admission to ICU	1.527	0.963 – 2.419
Preceding use of antibiotics	1.325	0.828 – 2.121
	**Prevalence**	
Department of admission<br>• internal medicine<br>• cardiothoracic surgery<br>• geriatrics	 12.34 %<br>10.05 %<br>15.07 %	
Place of residence<br>• own home<br>• nursing home<br>• rehabilitation facility	 12.01 %<br>23.81 %<br>13.89 %	

ICU = intensive care units

Table E. Influence of demographic factors and medical interventions on *de novo* ESBL-colonization during hospitalization at T1.

Factor	RR	95 % confidence intervals
Male sex	0.779	0.428 – 1.417
Surgical primary indication at admission	2.967	1.188 – 5.701
Antibiotic treatment	2.231	1.247 – 3.991
Antacid therapy	1.952	1.004 – 3.797
Other invasive procedures (invasive respiration, *i.v.* line, catheter, parenteral nutrition)	2.405	1.291 – 4.483
Surgical intervention	1.532	0.859 – 2.732
Diagnostic endoscopy	0.717	0.401 – 1.281

Table F. Influence of demographic factors and medical interventions on ESBL-persistence after 6 months after hospitalization (T2).

Factor	RR	95 % confidence intervals
Male sex	1.684	0.928 – 3.056
Surgical primary indication at previous hospitalization	1.694	0.971 – 2.958
Re-hospitalization during follow-up	1.058	0.545 – 2.053
Antibiotic therapy during follow up	0.824	0.184 – 3.683

Table G. Distribution of most abundant β-lactamases in the EPE species (all isolates n = 342).

Isolate	*Escherichia coli*	*Klebsiella pneumoniea*	*Klepsiella oxytoca*	*Citrobacter freundiiF*	*Enterobacter cloacea*
**CTX-M1/15**	258 (84.86 %)	18 (64.29 %)	1	2	0
**CTX-M9**	27 (8.89 %)	2 (7.14%)	2	0	1
**TEM**	151 (49.67 %)	16 (57.14 %)	0	3	0
**SHV**	6 (1.97 %)	20 (71.23 %)	0	0	0
**Total $n_{isolates}$**	304 (100 %)	2 (100 %)	3	3	4

Table H. Distribution of CTX-M1/15 and TEM and SHV β-lactamases in the EPEs (all isolates n = 342).

	TEM		SHV		
	**no**	**yes**	**no**	**yes**	**total**
**no CTX-M-1/5**	40 (63.49 %)	23 (36.51 %)	49 (77.78 %)	14 (22.22 %)	63 (100 %)
E. coli	29	17	40	6	46
K. pneumoniae	5	5	2	8	10
C. freundii	0	1	1	0	1
K. oxytoca	2	0	2	0	2
E. cloacae	4	0	4	0	4
**CTX-M-1/5**	132 (47.31 %)	147 (52.69 %)	267 (9.57 %)	12 (4.30 %)	279 (100 %)
E. coli	124	134	258	0	258
K. pneumoniae	7	11	6	12	18
C. freundii	1	0	1	0	1
K. oxytoca	0	2	2	0	2
E. cloacae	0	0	0	0	0

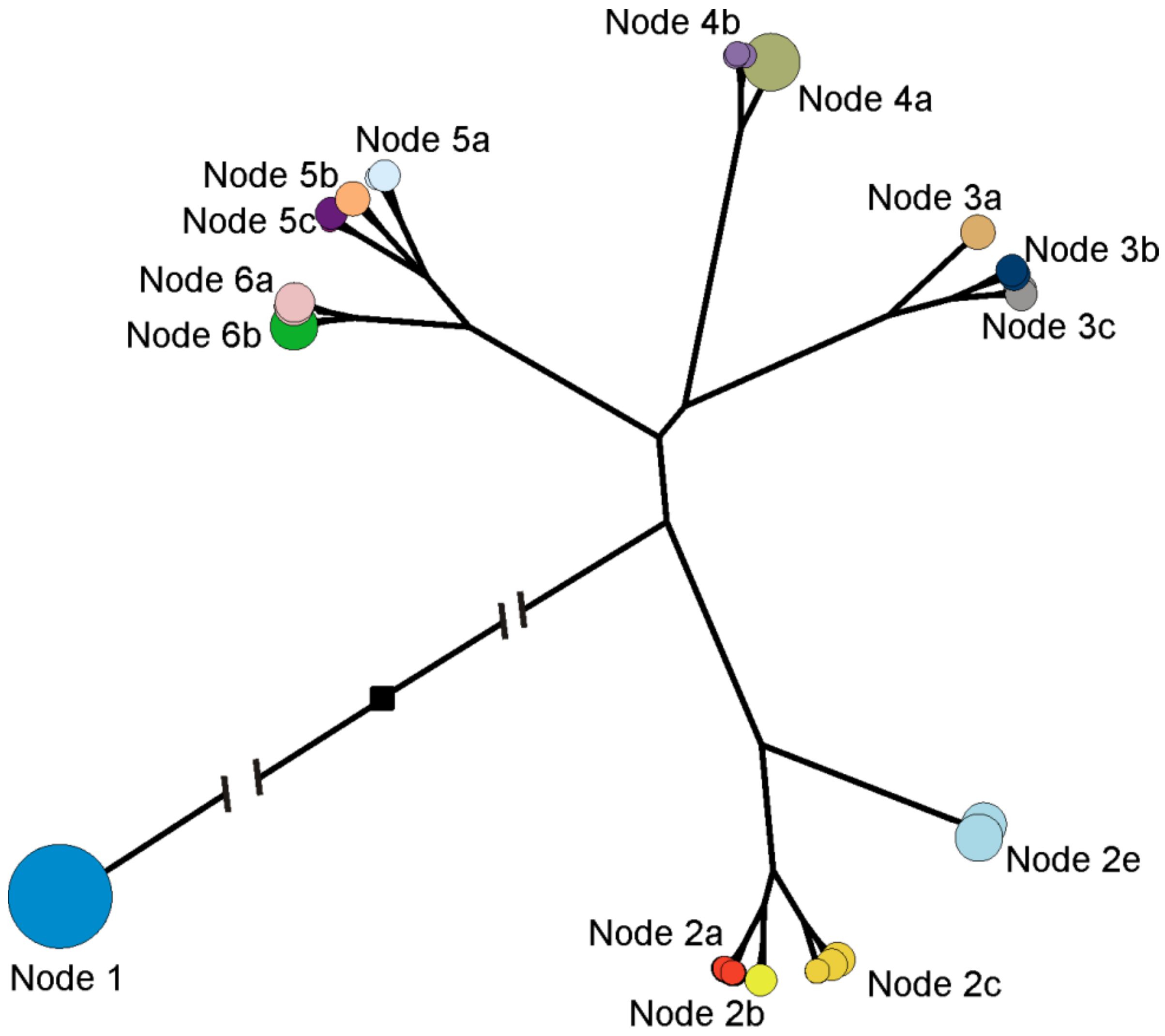

Fig A. Phylogeny of all EPE isolates based on the resistance gene profile determined using the hybridization microarray and applying the Pearson correlation and chi-square test. Analysis was performed using the BioNumerics 7.6 software (Applied Math NV, Sint-Martens-Latem, Belgium); Tree cluster analysis was performed using the Ward's method.

(S2 Figure in a separate file)

S2 Fig. Phylogeny of the *E. coli* isolates based on the ERIC-PCR band pattern. Analysis was performed applying the Dice similarity coefficient (0.5 % optimization, 1 % tolerance) using BioNumerics 7.6 software (Applied Math NV, Sint-Martens-Latem, Belgium). Tree construction was performed using the unweighted pair group method with arithmetic means (UPGMA).
