# Supplementary figures and images for: ESBL colonization and acquisition in a hospital population: The molecular epidemiology and transmission of resistance genes
Source: PLoS One. 2019 Jan 14;14(1):e0208505. doi: 10.1371/journal.pone.0208505 (PMC6331103; doi:10.1371/journal.pone.0208505)

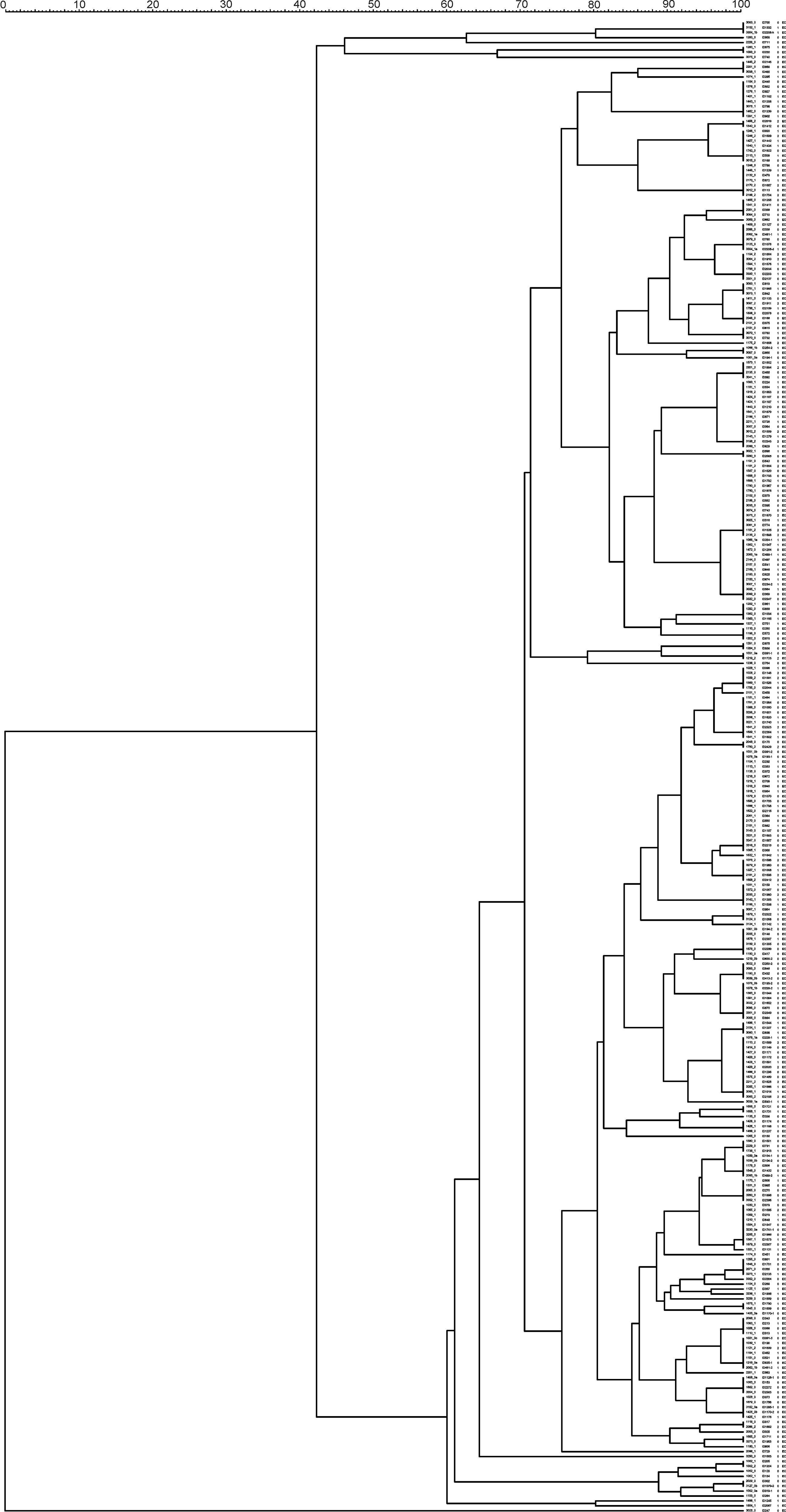

Supplement: S1 Fig — Analysis was performed applying the Dice similarity coefficient (0.5% optimization, 1% tolerance) using BioNumerics 7.6 software (Applied Math NV, Sint-Martens-Latem, Belgium). Tree construction was performed using the unweighted pair group method with arithmetic means (UPGMA). (JPG) [file pone.0208505.s003.jpg]
